# Ex-Situ Dual Hypothermic Oxygenated Machine Perfusion in Full-Left-Full-Right Split Liver Transplantation for Two Adult Recipients

**DOI:** 10.3390/jcm14186596

**Published:** 2025-09-19

**Authors:** Konrad Kobryń, Aleksandra Frankowska, Paweł Rykowski, Mateusz Bartkowiak, Andriej Zhylko, Marcin Morawski, Jan Stypułkowski, Łukasz Masior, Piotr Smoter, Waldemar Patkowski, Michał Grąt

**Affiliations:** Department of General, Transplant and Liver Surgery, Medical University of Warsaw, 02-091 Warsaw, Poland; aleksandrafrankowska01@gmail.com (A.F.); pawel.rykowski@wum.edu.pl (P.R.); mateusz.bartkowiak@wum.edu.pl (M.B.); andriej.zhylko@wum.edu.pl (A.Z.); marcin.morawski@wum.edu.pl (M.M.); jan.stypulkowski@wum.edu.pl (J.S.); lukasz.masior@wum.edu.pl (Ł.M.); piotr.smoter@wum.edu.pl (P.S.); waldemar.patkowski@wum.edu.pl (W.P.); michal.grat@wum.edu.pl (M.G.)

**Keywords:** split liver transplantation, dual hypothermic oxygenated machine perfusion, machine perfusion

## Abstract

**Background/Objectives**: The shortage of liver grafts remains a major challenge in transplantation. Full-left-full-right (FLFR) split liver transplantation (SLT) expands the donor pool by providing two grafts for small adult recipients. However, prolonged cold ischemia time (CIT) and ischemia-reperfusion injury (IRI) limit its success. **Methods**: We report a case of FLFR SLT utilizing ex situ dual hypothermic oxygenated machine perfusion (DHOPE) to mitigate IRI and enhance graft viability. A brain-dead donor’s liver was split under continuous DHOPE, followed by simultaneous transplantation into two adult recipients. **Results**: Both recipients exhibited stable graft function at one-year follow-up. DHOPE effectively reduced CIT and optimized postoperative recovery, with no major complications beyond Clavien–Dindo Grade IIIb. **Conclusions**: This is the first reported FLFR SLT using ex situ DHOPE for two adult recipients, demonstrating its feasibility in reducing CIT and improving outcomes. Machine perfusion may become a standard in FLFR SLT.

## 1. Introduction

The global demand for liver donor grafts continues to exceed the available supply, significantly limiting access to liver transplantation. Various strategies have been developed to address this shortage, including split liver transplantation (SLT), which is commonly employed in pediatric cases. In this approach, the left lateral segment (LLS) is transplanted into pediatric recipients, while the extended right lobe (ERL) is allocated to an adult recipient. However, smaller adult individuals often face greater challenges in obtaining appropriately size-matched grafts compared to average-sized adults, largely due to the limited global availability of smaller grafts. The full-left-full-right (FLFR) SLT technique enables the division of a whole liver into two grafts, which can be transplanted into two small adult recipients. This approach has the potential to reduce waiting times and waitlist mortality in this population.

One significant limitation of FLFR SLT, however, is the prolonged cold ischemia time (CIT) associated with the additional surgical dissection required, which has been correlated with adverse postoperative outcomes. The detrimental effects of prolonged CIT are particularly pronounced in FLFR SLT. Biliary complications represent a major concern, with rates ranging from 15 to 40% in split grafts compared to 10 to 15% in whole liver transplantation [1]. This disparity is partly attributable to the extended cold ischemia necessitated by the complex back-table splitting procedure, which typically adds 60–120 min to the preservation time [2].

Anatomical studies have demonstrated that biliary epithelial cells derive their blood supply exclusively from the peribiliary vascular plexus (PVP), which is fed primarily by the hepatic artery [3]. During cold ischemia, these delicate structures are particularly vulnerable to hypoxic injury, which manifests as biliary strictures, leaks, or cast formation post-transplantation. A multicenter analysis by Maggi et al. [4] demonstrated that each additional hour of CIT in split liver grafts was associated with a 1.2-fold increased risk of ischemic cholangiopathy (*p* = 0.003) and a 1.15-fold increased risk of graft loss within the first year (*p* = 0.008).

Traditionally, liver splitting is performed on the back table under static cold storage (SCS) conditions. Although in situ splitting can mitigate CIT by integrating the splitting process into the organ procurement phase, it substantially increases donor operative time by 60–90 min [5] and introduces several logistical challenges. In situ splitting requires the skill of masterclass surgeons trained both in transplant and liver surgery, substantially increases donor total operative time, and can create coordination challenges with donor hospitals. A survey by Bhangui et al. [6] revealed that only 28% of European transplant centers regularly perform in situ splitting, with the majority citing logistical constraints, including limited availability of qualified surgeons, extended donor operating room time, and coordination challenges with donor hospitals.

Ex situ dual hypothermic oxygenated machine perfusion (DHOPE) represents a promising strategy to address these limitations by minimizing CIT and ischemia-reperfusion injury (IRI). Hypothermic perfusion has emerged as a promising strategy for mitigating the challenges posed by prolonged CIT during complex liver transplantation procedures. Multiple mechanisms underlie its protective effects. End-ischemic hypothermic oxygenated perfusion has been shown to revitalize mitochondrial function through restoration of electron transport chain activity and ATP generation [7]. This process leads to decreased production of reactive oxygen species upon reperfusion, attenuating oxidative stress and inflammatory cascade activation [8]. The continuous circulation of preservation solution also facilitates washout of metabolic waste products that accumulate during static cold storage, improving microcirculation upon reperfusion [9].

In a randomized controlled trial, van Rijn et al. [10] demonstrated that dual hypothermic oxygenated machine perfusion significantly reduced ischemia-reperfusion injury compared to static cold storage, with lower peak transaminase levels and improved early allograft function. These benefits appear particularly relevant in the context of split liver transplantation, where the additional manipulation required exacerbates ischemia-reperfusion injury through direct mechanical trauma to the parenchyma [11].

By establishing dual oxygenated perfusion via the portal vein and hepatic artery under controlled hypothermic conditions, DHOPE has the potential to mitigate the risk of primary non-function and improve post-reperfusion outcomes, both of which are adversely affected by prolonged CIT and IRI. Herein, we present a case report detailing the technical execution of ex situ DHOPE in the context of FLFR SLT, culminating in the successful transplantation of both hemilivers into two adult recipients.

## 2. Materials and Methods

In July 2023, with the use of continuous dual hypothermic oxygenated perfusion, we performed a full-left-full-right graft split liver transplantation for two adult female recipients.

This study was conducted in accordance with the ethical standards of the Declaration of Helsinki of 1975 (revised in 2013). The transplantation procedures and associated research protocol were approved by the Institutional Review Board of the Medical University of Warsaw (approval number KB/6/2020, approved 13 January 2020). Written informed consent was obtained from both recipients for the split liver transplantation procedure using machine perfusion technology.

**Figure 1:**
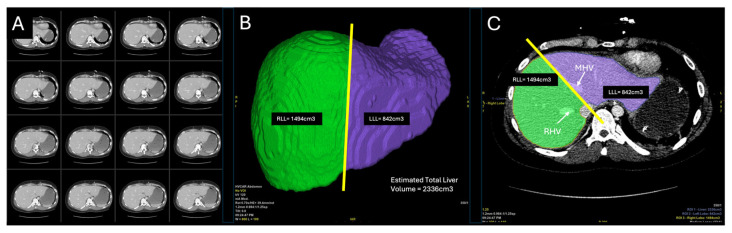
Preoperative liver evaluation and 3D reconstruction (total liver volume estimation). (**A**) Donor abdominal contrast enhanced computed tomography (CT). (**B**) Donor liver in CT following rendering and 3D reconstruction according to preoperative planning. Full-left-full-right (FLFR) division with estimated total liver volume (2336 cm^3^) and estimated volume for both lobes (RLL—right liver lobe, LLL—left liver lobe). (**C**) Preoperative estimated plane of division between RLL and LLL in correlation with right hepatic vein (RHV) and middle hepatic vein (MHV). RLL volume estimated at 1494 cm^3^ and LLL estimated at 842 cm^3^.

Prior to donor operation, the liver was evaluated in preoperative computed tomography (CT). A 3-dimensional liver model was reconstructed from the scans and anatomical vasculature was identified as feasible for splitting (Figure 1). An estimated volumetry of the graft was calculated at 2336 cm^3^. For a FLFR split, the volume of the left lobe was measured at 842 cm^3^ and the right lobe 1494 cm^3^, respectively. As standard practice at our center during CT reconstruction and volumetry, the dividing line between both grafts was established 1 cm to the right of the middle hepatic vein (MHV). The donor graft was retrieved at our hospital from a 22-year-old male who was declared brain-dead due to hydrocephalus. The donor had a body weight of 90 kg, height of 180 cm, and a body mass index (BMI) of 27.8 kg/m^2^ (Table 1). The calculated donor risk index was 1.72. The donor met our standard selection criteria for SLT, which include age > 21 years, body weight > 70 kg, BMI < 28 kg/m^2^, intensive care unit (ICU) stay < 7 days, serum sodium levels < 160 mEq/L, alanine aminotransferase (ALT) and aspartate aminotransferase (AST) levels < 3 times the normal range, hemodynamic stability, negative virology, hepatocyte macrosteatosis < 20%, and the absence of vascular anomalies in the liver confirmed by CT.

A multiorgan retrieval in a donation after brain death (DBD) approach was performed in its usual setting. In situ flushing during retrieval involved the use of StoreProtect Plus^®^ (CarnaMedica, Warsaw, Poland), a clinically optimized 4 °C University of Wisconsin (UW) preservation solution. Perfusion time was 15 min with total volume of 4000 mL delivered through the cannulated aorta and 1000 mL through the portal vein. Donor hepatectomy was completed within 45 min following cardiac arrest.

The graft was subsequently placed in 4 °C StoreProtect Plus^®^ (CarnaMedica, Warsaw, Poland) solution and transferred to the back table for preparation under SCS. The hepatoduodenal ligament was dissected and the portal trunk along with the common hepatic artery were identified, enabling further division.

**Figure 2:**
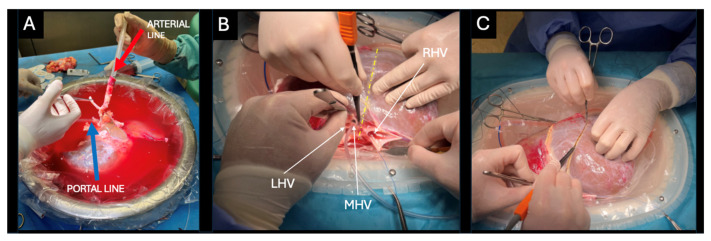
Back-table benching in static cold storage (SCS) conditions. (**A**) Fixation of cannulas. Red arrow points to the arterial line and blue arrow points to the portal line. The system must be sealed and checked for leaks. All collaterals need to be tied for dual hypothermic oxygenated perfusion (dHOPE) using the LiverAssist^®^ perfusion device. (**B**) Donor liver connected to the LiverAssist^®^ perfusion device undergoing dHOPE. The middle hepatic vein (MHV) is probed using a 10 fr cannula and a division of the hepatic veins is visible and marked by the yellow interrupted line. The right hepatic vein (RHV) remains with the inferior vena cava (IVC) and a 2 cm long stump common for the MHV and left hepatic vein (LHV). (**C**) Division along the marked Cantlie’s line with a small margin to the right of the middle hepatic vein. The donor liver is placed in the LiverAssist^®^ perfusion device in an orthotopic position mirroring similar conditions to that of the abdominal cavity.

Concurrently, the LiverAssist^®^ (Organ Assist^®^, Groningen, The Netherlands) machine perfusion device was assembled, and its circuit was primed with 2000 mL of UW machine perfusion solution. During the back-table procedure, the portal vein and supratruncal aortic patch containing the celiac trunk were cannulated with 24F cannulas (Figure 2A).

The graft was placed in the LiverAssist^®^ organ chamber, and both cannulas were connected to the perfusion system to re-establish vascular integrity. Throughout the splitting procedure, perfusion parameters were meticulously monitored and adjusted to maintain optimal conditions. Initial arterial perfusion was established at a pressure of 25 mmHg with a pulsatile flow rate of 150–200 mL/min (60 bpm). After completion of approximately 50% of the parenchymal transection, the arterial flow rate was gradually reduced to 100–150 mL/min while maintaining the pressure at 25 mmHg to compensate for the reduced parenchymal mass under perfusion. Portal venous pressure was consistently maintained at 5 mmHg, with initial flow rates of 400–500 mL/min that were similarly reduced to 300–350 mL/min as transection progressed.

Oxygenation was continuously maintained at 1000 mL/min with 100% O_2_ throughout the entire procedure. Perfusate samples were collected at 30 min intervals for blood gas analysis, with partial pressure of oxygen (pO_2_) consistently maintained above 600 mmHg to ensure adequate oxygen delivery. This approach aligns with the findings of Schlegel et al. [12], who demonstrated that maintaining pO_2_ > 500 mmHg is essential for supporting oxidative phosphorylation and ATP synthesis during hypothermic perfusion. The perfusion temperature was maintained at 10 °C, with additional cooling provided by optional ice.

The ex situ FLFR splitting procedure was conducted during continuous dual hypothermic oxygenated machine perfusion. A gauze pad was placed beneath the graft to elevate the plane of dissection, optimizing visualization. The anterior approach was applied resembling the orthotopic position of the liver in the abdominal cavity. The anterior approach employed during dHOPE splitting deserves specific consideration within the technical discourse. This approach, which resembles the orthotopic position of the liver, provides several advantages over posterior approaches during machine perfusion. First, it facilitates optimal visualization of the hilar structures and hepatic veins while maintaining stable perfusion dynamics. Anatomical orientation remains consistent with conventional in vivo splitting techniques, reducing the cognitive reorientation required by surgeons.

The left hepatic vein (LHV) and middle hepatic vein (MHV) were dissected and cut securing a 2 cm cuff for implantation of the left hemiliver (Figure 2B). The right hepatic vein (RHV) still attached to the inferior vena cava (IVC) remained with the right hemiliver. A sharp dissection of the liver hilum was performed up to the bifurcation of the left and right lobar structures. The left and right portal vein branches, hepatic arteries were isolated and looped, whereas the left and right hepatic ducts were first probed and then looped. The splitting plane was delineated along Cantlie’s line, approximately 1 cm to the right of the MHV (Figure 2C). Following full identification of the lobar structures and hilar plane, parenchymal transection was initiated using the Olympus SonoSurg™ G2 ultrasonic surgical system (Olympus Medical Systems Corp., Tokyo, Japan) with simultaneous ligation of vessels and bile ducts using bipolar electrocautery and/or Kelly clamps. Throughout the procedure, dual machine perfusion of the graft was maintained.

**Figure 3:**
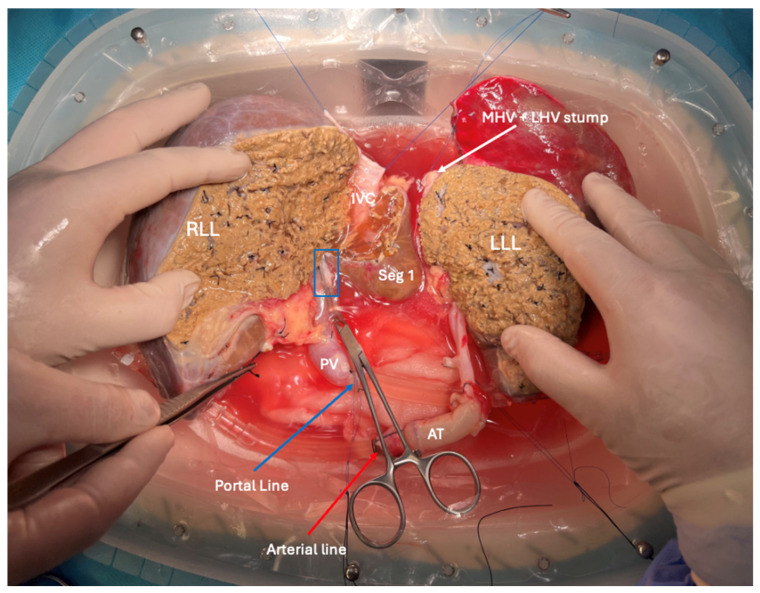
A completed ex situ full-left-full-right split procedure under continuous hypothermic oxygenated perfusion. At this stage, both grafts are under mono hypothermic oxygenated perfusion. The left liver lobe (LLL) is perfused via the arterial line (red arrow) inserted through the aortic trunk (AT). The right liver lobe (RLL) is perfused via the portal line (blue arrow) through the cannula inserted to the portal vein (PV). The Cooley Clamp is placed on the stump of the left portal branch (marked in the blue rectangular outline). The inferior vena cava (IVC) is separated from the MHV and LHV (white arrow).

Parenchymal transection was completed prior to dividing the looped hilar structures (Figure 3). The main trunk of the portal vein, the right hepatic artery, and the right hepatic duct were preserved within the full right graft. After division, the orifice of the left portal vein branch within the main portal trunk was closed with a running 6/0 polypropylene suture. The full right graft remained under machine perfusion and cooling via the portal line.

The left portal vein, the common hepatic artery with the celiac trunk and aorta, and the left hepatic duct were preserved within the full left graft, which was perfused and cooled via the arterial line. The left hepatic vein (LHV) and the middle hepatic vein (MHV), including its common orifice, were retained within the full left graft, while the inferior vena cava (IVC) and the right hepatic vein remained with the full right graft. The caudate lobe was preserved in the right graft but subsequently removed through resection. This decision was primarily driven by venous outflow optimization. The caudate lobe receives venous drainage through multiple small tributaries directly into the retrohepatic inferior vena cava (IVC), and preserving these connections would have necessitated either retaining a larger portion of the IVC (potentially complicating the left graft implantation) or extensive reconstruction of minute vessels [13]. Additionally, our preoperative volumetric assessment indicated that the right graft would provide adequate volume (GRWR 1.71) for the intended recipient even without the caudate contribution.

Venous tributaries of the MHV extending from segments V and VIII were retained in the right graft for subsequent reconstruction. The decision to reconstruct the V5 and V8 venous tributaries was based on their significantly larger caliber (8 mm and 10 mm, respectively) and the substantial parenchymal volume they drained, as revealed by preoperative CT volumetry, which estimated that approximately 30% of the right lobe outflow would be compromised without this reconstruction. According to Sano et al., venous tributaries larger than 5 mm in diameter draining significant portions of the anterior sector warrant reconstruction to prevent congestive outflow obstruction and potential graft dysfunction [14]. The continuous perfusion during dHOPE provided the advantage of immediate assessment of venous reconstruction patency and adequacy, allowing optimization before implantation.

Upon completing the split, the left hemiliver was the first to be removed from the LiverAssist^®^ device (Organ Assist^®^, Groningen, The Netherlands). It was immediately placed in static cold storage and handed over to the transplant team in the adjacent operating room, where the full left graft recipient’s procedure was underway. Following separation of the hemilivers, the left graft continued to receive arterial perfusion only (mono perfusion) at 25 mmHg, while the right graft received portal perfusion only at 5 mmHg. Flow rates were further adjusted to approximately 100 mL/min for the left graft arterial flow and 250 mL/min for the right graft portal flow to maintain stable pressure parameters. Temperature was consistently maintained between 8 and 10 °C throughout all phases of perfusion, with additional cooling provided by ice placed in the organ chamber when necessary to prevent temperature drift.

**Figure 4:**
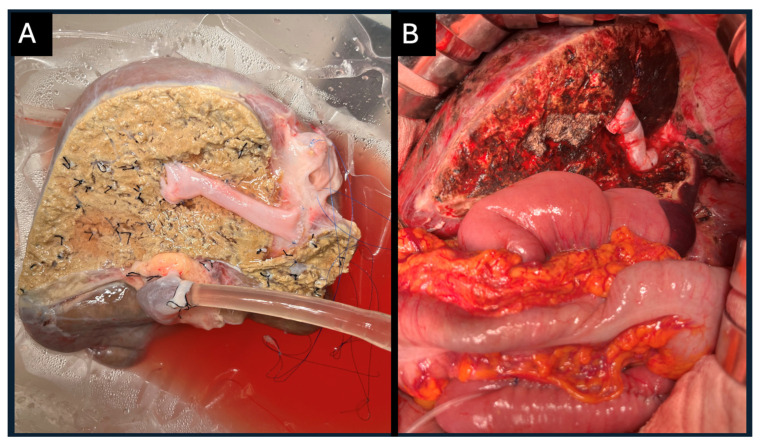
Full right liver graft. (**A**) Demonstrates segment V vein (V5) and segment VIII vein (V8) reconstruction to the inferior vena cava (IVC) using an iliac vein conduit from the same liver donor. Continuous mono hypothermic oxygenated perfusion during vascular graft reconstructions minimizes leaks ensuring vascular integrity before implantation. (**B**) Demonstrates the full right liver graft following implantation. All vascular anastomoses are complete. The V5 and V8 reconstruction to the IVC using a conduit drains from these two segments without complications. Hepatojejunal anastomosis is complete with a 6 fr drain placed at the anastomosis via a micro-jejunostomy.

The full right graft, remaining under machine perfusion, underwent additional venous reconstruction, including reconstruction of liver segment V vein (V5) and liver segment VIII vein (V8) orifices with the IVC using the donor’s femoral vein (Figure 4A). Continuous perfusion allowed to check for minor leaks from the reconstructed vessels minimizing blood loss and manipulations post reperfusion in the recipient (Figure 4B). Both transplantations were performed simultaneously at our transplant center.

## 3. Results

The left hemiliver recipient was a 64-year-old female, with an underlying disease primary biliary cholangitis (PBC), weighing 53 kg, with a height of 159 cm, body mass index (BMI) 21.0, and Model for End-Stage Liver Disease (MELD) score of 17. The recipient of the right graft was a 29-year-old female, undergoing liver transplantation due to hepatic alveolar echinococcosis, weighing 49 kg, with a height of 158 cm, BMI of 19.6, and MELD score of 9 (Table 2).

The retrieved donor liver weighed 1940 g. After splitting, the left hemiliver weighed 569 g and the right hemiliver 840 g, resulting in graft-to-recipient weight ratios (GRWR) of 1.07 and 1.71, respectively. Benching of the graft at the back table prior to DHOPE perfusion required 60 min.

### 3.1. Full Left Graft

The total cold ischemia time (CIT) for the full left graft was 324 min, comprising 210 min of DHOPE perfusion, 8 min of mono (arterial only) hypothermic oxygenated machine perfusion and 106 min of static cold storage (SCS). The concept of “functional CIT” distinguishes machine perfusion time from static cold storage, as machine perfusion provides continuous oxygenation and nutrient delivery, mitigating many deleterious effects of conventional cold ischemia [15]. Functional CIT was reduced to 114 min (Table 3), representing a reduction of 65% compared to the total CIT.

The graft’s left hepatic vein (LHV) and middle hepatic vein (MHV) were anastomosed to a 2 cm-long cuff of the native LHV and MHV using 4/0 polypropylene running suture. Portal vein anastomosis was performed end-to-end between the recipient’s portal vein and the graft’s left portal branch using a continuous 5/0 polypropylene suture. Arterial anastomosis was completed with a Carrel patch of the graft’s celiac trunk and the recipient’s hepatic artery bifurcation. The biliary anastomosis was carried out via a hepatojejunal Roux-en-Y loop using interrupted 6/0 polydioxanone sutures. The recipient required an intraoperative transfusion of 2 units of packed red blood cells (PRBC) and 3 units of fresh frozen plasma (FFP). Simultaneous arterial and venous reperfusion was achieved without post-reperfusion syndrome (PRS). Due to the smaller mass of the left graft, the recipient’s splenic artery was ligated to modulate portal flow.

Postoperative laboratory findings revealed peak levels of aspartate aminotransferase (AST) and alanine aminotransferase (ALT) on postoperative day 2 (POD 2), with values of 909 U/L and 1172 U/L, respectively. These levels normalized by POD 7, reaching 56 U/L (AST) and 157 U/L (ALT), total serum bilirubin (TBIL) of 7.03 mg/dL, and the international normalized ratio (INR) was 1.17. An abdominal ultrasound (US) carried out on POD 7 revealed a 91 × 96 mm fluid collection at the graft’s transection plane, a cholangiogram later confirmed a biloma. Percutaneous transhepatic biliary drainage (PTBD) with external–internal biliary catheter placement was performed six days later. On POD 22, an enteroscopy confirmed a persistent anastomotic biliary fistula. A relaparotomy was performed on POD 25 necessitating a redo hepatojejunal anastomosis. The surgery proved to be successful. The patient was discharged on POD 47 in good general condition.

### 3.2. Full Right Graft

Total cold ischemia time (CIT) for the full right graft was 440 min, comprising 210 min of dual hypothermic oxygenated machine perfusion (DHOPE) and 80 min of mono (portal-only) hypothermic oxygenated machine perfusion. Functional CIT was reduced to 150 min, representing a 66% reduction from the total CIT.

Following the removal of the left graft from the LiverAssist^®^ device (Organ Assist^®^, Groningen, The Netherlands), an additional 80 min was required for venous reconstruction and drainage of segments V (V5) and VIII (V8) to the IVC. The right hemiliver was then implanted into the recipient. Overall time of static cold storage (SCS) was 105 min (Table 3).

The full right graft was implanted with IVC replacement and end-to-end anastomosis using a 4/0 polypropylene suture. An end-to-end portal vein anastomosis was performed using a continuous 5/0 polypropylene suture. Arterial anastomosis was carried out using an end-to-end technique between the graft’s right hepatic artery and the recipient’s proper hepatic artery with a running 7/0 polypropylene suture. The biliary anastomosis was performed via a hepatojejunal Roux-en-Y loop using interrupted 5/0 polydioxanone sutures. The recipient required intraoperative transfusion of 3 units of packed red blood cells (PRBC) and 2 units of fresh frozen plasma (FFP). No post-reperfusion syndrome (PRS) was observed.

Postoperatively, the recipient exhibited a peak aspartate aminotransferase (AST) level of 2327 U/L on postoperative day 1 (POD 1) and a peak alanine aminotransferase (ALT) level of 3205 U/L on POD 2. By POD 7, these values had decreased to 40 U/L (AST) and 257 U/L (ALT). On POD 7, total serum bilirubin (TBIL) stabilized at 0.92 mg/dL with an international normalized ratio (INR) of 1.18. Abdominal ultrasound (US) performed on POD 7 revealed a fluid collection measuring 102 × 15 mm adjacent to segments V and VIII of the transection plane. On POD 8, a biliary fistula did not present itself during a cholangiogram at that time.

On POD 15, due to recipients’ abdominal signs, a laparotomy was performed, a biloma was confirmed and removed. The anastomotic fistula was identified, and an additional suture was placed at the biliary anastomosis and the abdomen flushed with saline and drained. Two days later (POD 17), a percutaneous transhepatic biliary drainage (PTBD) was performed as means of decompressing the bile duct for better healing. On POD 29, another relaparotomy was required due to a persistent biliary fistula. A re-do hepaticojejunal anastomosis was performed. On POD 36, a control cholangiogram identified an anastomotic leak and on POD 37 a percutaneous transhepatic biliary drainage (PTBD) was carried out. This proved successful, no further interventions were required and PTBD was terminated on POD 59. The patient was discharged from hospital on POD 70 and remains in good general condition.

At 18 months post SLT, both recipients are in good general condition with stable liver function and did not require readmission to hospital.

In the context of split liver transplantation, various preservation techniques offer distinct advantages and limitations. Table 4 summarizes the key differences between static cold storage (SCS), dual hypothermic oxygenated perfusion (dHOPE), and normothermic machine perfusion (NMP).

## 4. Discussion

With the ongoing shortage of liver donors worldwide, it is crucial to explore strategies that augment the existing graft reservoir. Split liver transplantation has proven to be an effective approach for expanding the graft pool, successfully reducing both the waiting list and waitlist mortality among pediatric and small adult recipients. This is particularly relevant given that only 0.5–2% of deceased donor livers in Europe are currently used for FLFR splitting according to recent European Liver Transplant Registry data [16].

Our decision to employ dHOPE rather than NMP was based on several considerations. While NMP offers superior functional assessment capabilities by mimicking physiological conditions [17], it necessitates continuous, uninterrupted perfusion and carries the risk of additional hepatic injury during potential recooling phases if machine failure occurs [18]. In contrast, dHOPE provides a favorable balance between metabolic support and logistical flexibility, particularly advantageous in the complex setting of full-left-full-right splitting [19]. Furthermore, dHOPE has demonstrated efficacy in reducing oxidative stress and promoting mitochondrial recovery, which is particularly relevant in grafts subjected to the additional manipulation involved in splitting procedures [20].

Although no studies have definitively proven the superiority of dHOPE over HOPE in preserving biliary function, our decision to employ dHOPE was guided by several theoretical mechanisms. The biliary epithelium receives its blood supply primarily through the peribiliary vascular plexus (PVP), which derives predominantly from the hepatic artery. Hypothetically, the addition of arterial perfusion in dHOPE provides direct oxygenation to these vulnerable biliary structures, potentially reducing ischemic cholangiopathy—a particularly concerning complication in split liver transplantation. Schlegel et al. demonstrated in their systematic review that end-ischemic dual hypothermic oxygenated perfusion reduced biliary complications in donation after circulatory death (DCD) grafts, suggesting potential benefit in other high-risk settings such as SLT [21]. Furthermore, Brüggenwirth et al. showed that the peribiliary vascular plexus maintains better integrity with dual perfusion compared to portal perfusion alone [22]. Given that biliary complications remain the Achilles’ heel of split liver transplantation, with rates ranging from 15 to 40% according to Noujaim et al. [23], we prioritized dHOPE as a mitigation strategy despite the absence of definitive comparative evidence to HOPE.

Our data demonstrated a substantial reduction in functional cold ischemia time through the implementation of dHOPE. The concept of “functional CIT” distinguishes machine perfusion time from static cold storage, as machine perfusion provides continuous oxygenation and nutrient delivery, mitigating many deleterious effects of conventional cold ischemia [24]. In the present study, the functional CIT was reduced to 114 min for the left graft and 150 min for the right graft, compared to the total CIT of 324 and 440 min, respectively, representing reductions of 65% and 66%. These reduction rates align with findings from similar studies: Czigany et al. reported functional CIT reductions of 40–60% in their series of hypothermic machine perfusion cases [25], while Jochmans et al. observed a median functional CIT reduction of 53% in their multicenter trial [26]. The concept of functional CIT reduction is particularly relevant in the context of split liver transplantation, where conventional total CIT often exceeds acceptable thresholds due to extensive back-table manipulation. By metabolically supporting the graft during the splitting procedure, dHOPE effectively converts a significant portion of the otherwise harmful ischemic time into a controlled preservation period.

Regarding the anterior approach and comparison with Broering’s technique, the anterior approach employed during dHOPE splitting deserves specific consideration within the technical discourse. This approach, which resembles the orthotopic position of the liver, provides several advantages over posterior approaches during machine perfusion. First, it facilitates optimal visualization of the hilar structures and hepatic veins while maintaining stable perfusion dynamics. Anatomical orientation remains consistent with conventional in vivo splitting techniques, reducing the cognitive reorientation required by surgeons. Although no formal comparative studies between anterior and posterior approaches during machine perfusion exist, Yoon et al. demonstrated in their series of conventional ex situ splits that the anterior approach was associated with shorter parenchymal transection times and reduced bleeding upon reperfusion [27]. Regarding venous outflow, our technique differs from that described by Broering et al., who advocated splitting the vena cava and middle hepatic vein to preserve venous drainage for both grafts [28]. We opted instead to retain the IVC with the right lobe while maintaining the MHV with the left lobe, subsequently reconstructing V5 and V8 drainage in the right lobe using venous grafts. Our approach was informed by three considerations: first, the continuous machine perfusion allowed for immediate testing of venous reconstruction integrity, addressing potential leaks before implantation; second, this technique preserves the option of conventional caval replacement or piggyback techniques for the right graft based on recipient considerations; third, no further reconstruction is required for the left lobe. This approach also differs from that recently described by Cillo et al., who implemented Broering’s technique of splitting both the MHV and IVC along a plane that provides each hemiliver with its respective venous drainage territory [29]. Based on our experience with living donor liver transplantation, our method is more acquainted in our setting and offers greater flexibility in the final implantation strategy. We strongly feel it is enough to compromise one lobe (right) with vascular reconstructions while reducing CIT for the other.

The full right graft was implanted with IVC replacement and end-to-end anastomosis using a 4/0 polypropylene suture. This approach was chosen for several reasons. First, the reconstruction of V5 and V8 venous tributaries directly onto the donor IVC created a complex venous outflow system that was optimally preserved by maintaining the structural integrity of the donor IVC. Transecting and reimplanting these reconstructed veins onto the recipient’s preserved IVC would have introduced additional anastomoses and increased the risk of outflow compromise. Second, the recipient’s underlying condition (hepatic alveolar echinococcosis) often involves retrohepatic IVC invasion or proximity, making complete IVC replacement a safer oncological approach in selected cases [15]. Although preoperative imaging did not demonstrate definitive IVC involvement, the intraoperative assessment revealed perivenous inflammatory changes that warranted caution. The venous reconstructions were performed during continuous hypothermic perfusion, with both V5 (8 mm diameter) and V8 (10 mm diameter) tributaries anastomosed to openings created in the donor IVC using iliac vein conduits from the same donor. This approach maintained a straight outflow tract with minimal angulation, optimizing hemodynamics. The dHOPE setting allowed for real-time assessment of reconstruction patency and identification of potential leaks prior to implantation.

The assertion that ex situ splitting using only a portal vein cannula might be safer than dHOPE deserves contextualization. This perspective, advocated by Mabrut et al., stems primarily from concerns about hepatic artery injury during cannulation and perfusion. Indeed, the hepatic artery’s smaller caliber, fragility, and propensity for vasospasm make arterial cannulation technically more challenging than portal cannulation [30]. However, this theoretical risk must be balanced against the potential benefits of arterial perfusion, particularly in the context of biliary preservation.

The relative safety of portal-only versus dual perfusion likely depends on specific donor-recipient factors. In cases with extended predicted cold ischemia time, abnormal hepatic arterial anatomy, or recipients with risk factors for ischemic cholangiopathy, the benefits of dual perfusion may outweigh the technical risks. Conversely, in scenarios with shorter anticipated preservation times or when arterial cannulation proves technically challenging, portal-only perfusion represents a reasonable alternative. Eshmuminov et al. demonstrated in their comparative analysis that while dual perfusion provided superior metabolic support, portal-only perfusion still offered significant advantages over static cold storage in terms of ATP preservation and mitochondrial function [31].

In our case, the decision to employ dHOPE reflected our center’s extensive experience with dual cannulation techniques, the complex nature of FLFR splitting, and our prioritization of optimal biliary preservation. We suggest that perfusion strategy selection should consider center expertise, technical feasibility, and case-specific risk assessment rather than adhering to a one-size-fits-all approach.

Two cases illustrating the use of dual hypothermic oxygenated perfusion (DHOPE) in split liver transplantation (SLT) have been described in the literature. Both cases involved pediatric recipients, with either full or hyper-reduced left lateral segments (LLS) transplanted into the child and extended right lobes (ERL) into small adults [32,33]. These studies demonstrated the effectiveness of DHOPE in grafts with anticipated prolonged cold ischemia time (CIT), yielding favorable postoperative outcomes without device-related complications in any of the recipients. Spada et al. reported a CIT reduction to 11 h for LLS and 14 h for ERL [34], while Thorne et al. observed a shortening of CIT to less than 4 h for LLS and less than 8 h for ERL, accounting for additional static cold storage (SCS) time required during back-table preparation and transport to the second transplant center [35]. In our setting of a local organ procurement and local transplants, dHOPE allowed for a definite reduction in functional CIT of 2.5 h vs. <5 h for the full right graft and <2 h vs. <4 h for the full left graft.

At the time of preparing this manuscript, a single case report of full-left-full-right (FLFR) split liver transplantation (SLT) during hypothermic oxygenated perfusion (HOPE) has previously been described [34]. In that case, the left graft was transplanted into a 4-year-old pediatric recipient, and the right graft was transplanted into a 38-year-old adult. The study demonstrated early recovery of hepatic function with normal liver biochemistry at a six-month follow-up. In 2024, another report was published by Cillo et al. who transplanted the left hemiliver to a 7-year-old boy and the right hemiliver to a 64-year-old adult [29]. The authors demonstrate the adoption of splitting the inferior vena cava along the middle hepatic vein introduced by Broering during dHOPE as a feasible and safe FLFR split technique with significant reduction of cold ischemia time. Our case study gives clear evidence that implementation of dHOPE for split liver transplantation may even shorten CIT by 50%.

Despite the implementation of dHOPE, both recipients experienced biliary complications requiring surgical revision. These findings warrant critical examination of bile duct perfusion adequacy during the splitting procedure. During conventional liver procurement, the biliary epithelium receives dual blood supply—both from the hepatic artery via the peribiliary vascular plexus (PVP) and from portal venous branches [3]. The splitting procedure invariably disrupts small collateral vessels between these systems at the transection surface. While dHOPE provided continuous perfusion via both the hepatic artery and portal vein, the perfusion pattern likely differs from physiological conditions, particularly at the hilar plate where bile ducts are most vulnerable.

Additionally, the parenchymal transection inherently creates a plane of injury with exposed bile ductules that may leak bile postoperatively, contributing to periductal inflammation and subsequent stricture formation. Hansen et al. [36] demonstrated using fluorescence angiography that perfusion at the cut surface during SLT can be suboptimal despite macroscopically adequate global perfusion. Our experience suggests that while dHOPE may mitigate ischemic injury to the biliary system, it does not eliminate the technical challenges associated with bile duct division and reconstruction in FLFR SLT.

The persistence of biliary complications despite dHOPE implementation requires multifaceted consideration. While machine perfusion offers theoretical advantages for biliary preservation, several factors may contribute to the development of biliary complications in this setting. First, the technical aspects of bile duct division and reconstruction remain critically important. The biliary anastomosis in split liver transplantation often involves smaller ducts with reduced blood supply at their distal ends, regardless of preservation method [37]. Our technique involved hepatojejunal Roux-en-Y anastomosis for both grafts, which while providing reliable long-term results, carries an initial higher risk of leakage compared to duct-to-duct reconstruction [38].

Second, the efficacy of perfusion in preserving biliary viability depends on multiple parameters beyond the simple presence of flow. Brüggenwirth et al. [22] demonstrated that pressure dynamics, oxygen tension, and composition of the perfusate all influence bile production and biliary epithelial integrity during machine perfusion. The optimal parameters specifically for split liver grafts remain undefined, with most current protocols extrapolated from whole liver perfusion studies.

Third, biliary complications in split liver transplantation likely represent a multifactorial problem where preservation strategy is just one component. Watson et al. [18] proposed that biliary complications result from the intersection of donor factors (e.g., age, steatosis), technical factors (e.g., duration of warm ischemia during splitting, precision of hilar dissection), and recipient factors (e.g., postoperative hemodynamics, immunosuppression regimen). This complexity may explain why single interventions, including advanced preservation techniques, show inconsistent results in eliminating biliary morbidity in split liver transplantation. Until now, posttransplant biliary complications remain the Achilles Heel. Further studies will show if the use of machine perfusion during split liver transplantation will bring a significant reduction in these complications.

Liver splitting can be performed either using an ex situ technique on the back table or in situ prior to organ procurement, like the procedure used in living donor operations. In situ splitting in a hemodynamically stable cadaveric donor is generally considered safer than ex situ splitting, as it minimizes CIT, reduces biliary complications, and lowers the risk of primary graft non-function, despite requiring a longer donor operation [39]. However, a notable drawback is the necessity for two specialist surgeons with expertise in hepatobiliary surgery. In contrast, the ex situ technique is more accessible in most healthcare settings in Poland. It is logistically simpler to perform, as it does not require the donor recovery team to have extensive experience in liver surgery. Liver splitting during machine perfusion combines the advantages of both ex situ and in situ approaches. This strategy enhances the logistical efficiency of the procedure while leveraging the benefits of continuous perfusion, leading to shorter CIT and a reduced risk of early allograft dysfunction (EAD). In our study, no complications greater than Grade IIIb, based on the Clavien–Dindo Classification, were observed in these transplant recipients. Postoperative outcomes of the ex situ technique are comparable to those of the in situ approach.

Following the routine procedure of back-table benching as it is for whole graft transplantation, the donor liver once connected to dual hypothermic oxygenated perfusion can be split to a point where each graft will continuously be perfused with hypothermic oxygenated fluid via a single line—this enhanced safety. An anterior approach for splitting the parenchyma during dHOPE seems more natural for liver surgeons, mainly due to its orthotopic and stabilized position in the basin. The cannulas are fixated, thus the chances of insufficient perfusate flow via them is less probable. The many scenarios and vast usage of dHOPE in FLFR SLT is still to be discovered, but it is not uncommon that anatomical variations cause one graft to be more demanding in preparation from the other. Now surgeons can better focus their attention on the more difficult graft allowing the easier one to wait its turn. Technically, there is enough space in the LiverAssist^®^ basin to keep both grafts under continuous perfusion and carry on with the venous reconstruction of the full right graft. Although the full left graft usually, after completing the parenchymal division, is ready to be handed over to the transplant team, in case of recipient hepatectomy difficulties and prolonging time, it can easily continue mono HOPE.

Performing a split procedure during continuous normothermic perfusion (NMP) has been explored, initially as a proof of concept and, more recently, in clinical practice. Krendl et al. investigated the 90-day outcomes of NMP in right and extended right graft recipients, reporting 100% patient and graft survival. However, 50% of patients (3 out of 6) required early relaparotomy, and 33.3% (2 out of 6) experienced postoperative biliary complications [37]. Unlike graft preservation in hypothermia, NMP mimics physiological body conditions, allowing for preoperative assessment of hepatic viability and liver synthetic function. This approach is particularly beneficial in high-risk donors where hepatic function may be uncertain. However, in our case, pretransplant functional hepatic assessment was unnecessary.

One key drawback of NMP is the potential need for detrimental recooling in the event of unintended machine perfusion cessation or during transport to another transplant center. In contrast, graft splitting under hypothermic perfusion avoids this issue. The transition from dHOPE to SCS eliminates the need for a recooling phase, thereby reducing the risk of additional hepatic injury caused by sudden temperature changes.

### Limitations

Several limitations of this study warrant acknowledgment. First, as a single case report, our findings cannot be generalized to all FLFR SLT scenarios. The successful outcomes observed may reflect not only the benefits of dHOPE but also favorable donor and recipient characteristics, surgical expertise, and post-transplant management protocols. Second, while we did not observe device-related complications, potential risks exist including hypothermia-induced endothelial injury [37], inadequate perfusion leading to heterogeneous preservation, and mechanical issues such as pressurization failures or gas embolism. Third, our functional CIT calculations represent a theoretical construct that requires validation through larger studies with biomarker and metabolic profiling during perfusion. Fourth, although both recipients exhibited good outcomes at 18-month follow-ups, the biliary complications encountered highlight that machine perfusion alone may not eliminate all challenges associated with FLFR SLT. Finally, the cost-effectiveness of dHOPE in the FLFR SLT setting remains to be established, as the additional resources required must be balanced against potential benefits in graft function and recipient outcomes.

To address these limitations and advance the field, we propose several future research directions. Multicenter randomized controlled trials comparing dHOPE versus HOPE in FLFR SLT are essential to establish the optimal perfusion strategy. Such studies should include standardized surgical techniques, perfusion parameters, and outcome measures to enhance comparability across centers. Additionally, investigation of dHOPE’s role in expanding the donor pool for FLFR SLT to include marginal donors (e.g., older donors, steatotic grafts) represents an important frontier. Mergental et al. demonstrated, in a pilot study, that machine perfusion enabled successful utilization of extended criteria donors for standard liver transplantation; whether similar benefits apply to split liver transplantation requires focused investigation [38].

## 5. Conclusions

Our case study demonstrates the feasibility and potential benefits of continuous dHOPE in FLFR split liver transplantation, particularly in scenarios with anticipated prolonged CIT. Compared to SCS, dynamic cold preservation has demonstrated the ability to reduce CIT, mitigate the severity of IRI, and improve logistical efficiency. The implementation of this technique resulted in substantial functional CIT reduction, with both recipients achieving stable graft function at 18-month follow-ups despite early biliary complications requiring intervention.

These findings have several important clinical implications for surgical decision-making in SLT. First, they suggest that machine perfusion may expand the feasibility of FLFR splitting by mitigating the deleterious effects of prolonged cold ischemia. Second, our experience indicates that the anterior approach for ex situ splitting during continuous perfusion is technically feasible and may offer advantages in terms of anatomical orientation and real-time assessment of vascular reconstruction.

Looking forward, several research directions warrant exploration. Prospective studies comparing dHOPE, HOPE, and NMP in the context of FLFR SLT are needed to establish the optimal perfusion strategy. Additionally, investigation of perfusate biomarkers that predict biliary complications could enable the development of tailored approaches to post-splitting preservation [40]. Machine perfusion may also facilitate extended criteria donor utilization for FLFR SLT, potentially expanding this underutilized approach in European centers where current practices remain conservative due to concerns about splitting-related complications [41].

In conclusion, we believe that machine perfusion technologies represent a significant advancement in FLFR split liver transplantation, with the potential to increase the utilization of this technique and thereby expand the donor pool for adult recipients. Continuous refinement of both technical aspects and selection criteria will be essential to optimize outcomes and establish machine perfusion as the standard of care in FLFR SLT.

## Figures and Tables

**Table 1:** Donor characteristics.

Donor Characteristics	Value
Age (years)	22
Gender	Male
Weight (kg)	90
Height (cm)	180
Body Mass Index (kg/m^2^)	27.8
Risk Index	1.72
Estimated Total Liver Volume (cm^3^)	2336
Estimated Right Liver Lobe Volume (cm^3^)	1494
Total Donor Liver Weight (g) ex situ	1940

**Table 2:** Recipient demographics.

Recipient Demographics	Left Graft	Right Graft
Age (years)	64	29
Gender	Female	Female
Weight (kg)	53	49
Height (cm)	159	158
BMI (kg/m^2^)	21.0	19.6
MELD score	17	9
Underlying disease	PBC	Hepatic Alveolar
		Echinococcus

BMI, body mass index; MELD, model for end-stage liver disease; PBC, primary biliary cholangitis.

**Table 3:** Full left and full right graft characteristics.

Graft Feature	Full Left Liver Graft	Full Right Liver Graft
Preoperative Estimated Graft Volume (cm^3^)	842	1494
Graft Weight (g)	569	840
GRWR	1.07	1.71
Total CIT (min)	324	440
Functional CIT	114	150
Machine Perfusion Time (min)	210 (dual) + 8 (mono)	210 (dual) + 80 (mono)
Static cold storage (min)	106	105
Vascular and Biliary structures retained	LHV, MHV, Left PV, Celiac Trunk, Left Hepatic Duct	RHV, IVC, Right PV, RHA, Right Hepatic Duct

GRWR, graft-to-recipient weight ratio; CIT, cold ischemia time, LHV, left hepatic vein; MHV, middle hepatic vein; PV, portal vein; RHV, right hepatic vein; IVC, inferior vena cava; RHA, right hepatic artery.

**Table 4:** Differences between various preservation techniques (static cold storage vs. dual hypothermic oxygenated perfusion vs. normothermic machine perfusion).

Feature	Static Cold Storage (SCS)	Dual Hypothermic Oxygenated Perfusion (dHOPE)	Normothermic Machine Perfusion (NMP)
Temperature	4 °C	10 °C	37 °C
Metabolism	Anaerobic	Reduced aerobic	Aerobic
ATP production	Minimal	Intermediate	Maximal
Graft assessment	Not possible	Limited	Comprehensive functional assessment
Biliary protection	Poor	Enhanced via dual perfusion	Enhanced with functional assessment
Logistical flexibility	High	Moderate	Limited (requires constant monitoring)
Risk upon device failure	Low	Moderate	High (requires recooling)
Technical complexity	Low	Moderate	High
Recooling need if interrupted	No	No	Yes (potential additional injury)
Evidence in FLFR SLT	Established	Emerging	Limited

## Data Availability

No new data were created or analyzed in this study.

## Abbreviations

The following abbreviations are used in this manuscript:

ATP	Adenosine Triphsphate
ALT	Alanine Aminotransferase
AST	Aspartate Aminotransferase
BMI	Body Mass Index
CIT	Cold Ischemia Time
CT	Computed Tomography
DBD	Donation After Brain Death
dHOPE	Dual Hypothermic Oxygenated Perfusion
EAD	Early Allograft Dysfunction
ERL	Extended Right Lobe
FLFR	Full-Left-Full-Right
FFP	Fresh Frozen Plasma
GRWR	Graft-to-Recipient Weight Ratio
HOPE	Hypothermic Oxygenated perfusion
ICU	Intensive Care Unit
IRI	Ischemia-Reperfusion Injury
IVC	Inferior Vena Cava
LLS	Left Lateral segment
LHV	Left Hepatic Vein
MELD	Model for End-Stage Liver Disease
MHV	Middle Hepatic Vein
NMP	Normothermic Machine Perfusion
PBC	Primary Biliary Cholangitis
POD	Postoperative Day
PRBC	Packed Red Blood Cells
PRS	Post-Reperfusion Syndrome
PTBD	Percutaneous Transhepatic Biliary Drainage
PV	Portal Vein
ROS	Reactive Oxygen Species
RHA	Right Hepatic Artery
RHV	Right Hepatic Vein
SCS	Static Cold Storage
SLT	Split Liver Transplantation
TBIL	Total Serum Bilirubin
US	Ultrasound
UW	University of Wisconsin (preservation solution)
V5	Segment V Vein
V8	Segment VIII Vein

## References

[1] Hessheimer A.J., Coll E., Torres F., Ruíz P., Gastaca M., Rivas J.I., Gómez M., Sánchez B., Santoyo J., Ramírez P. (2019). Incidence, risk factors, and outcomes of biliary complications after adult split liver transplantation: A systematic review and meta-analysis. Am. J. Transplant..

[2] Vagefi P.A., Hirose R., Pomfret E.A., Freise C.E., Ascher N.L., Roberts J.P., Emond J.C., Brown R.S., Terrault N.A., Rosenthal P. (2013). Ex situ split liver transplantation: Current status. Curr. Opin. Organ. Transplant..

[3] op den Dries S., Karimian N., Sutton M.E., Westerkamp A.C., Nijsten M.W.N., Gouw A.S.H., Wiersema-Buist J., Lisman T., Leuvenink H.G.D., Porte R.J. (2016). Protection of bile ducts in liver transplantation: Looking beyond ischemia. Transplantation.

[4] Maggi U., Andorno E., Rossi G., De Carlis L., Colledan M., Salizzoni M., Risaliti A., Baccarani U., Bresadola V., Lenci I. (2018). Risk factors for biliary complications after liver transplantation: A European multicenter study. Liver Transpl..

[5] Lauterio A., Di Sandro S., Concone G., Buscemi V., Spada M., Alfani A., De Carlis R., Benuzzi L., Danieli M., Bongini M. (2019). Current status and perspectives in split liver transplantation. World J. Gastroenterol..

[6] Bhangui P., Petrowsky H., Nemes B., Jochmans I., Cillo U., Klempnauer J., Settmacher U., Ortiz de Urbina J., Vennarecci G., Parente A. (2022). Current status of full-right-full-left split liver transplantation in Europe: Results of an international survey. Liver Transpl..

[7] Schlegel A., Muller X., Kalisvaart M., Muellhaupt B., Perera M.T.P.R., Isaac J.R., Clavien P.A., Muiesan P., Dutkowski P., Graf R. (2016). Hypothermic oxygenated perfusion (HOPE) downregulates the immune response in a rat model of liver transplantation. Ann. Surg..

[8] Czigany Z., Lurje I., Tolba R.H., Neumann U.P., Tacke F., Lurje G., Bednarsch J., Roderburg C., Demir M., Kroy D.C. (2020). Ischemia-reperfusion injury in marginal liver grafts and the role of hypothermic machine perfusion: Molecular mechanisms and clinical implications. J. Clin. Med..

[9] Boteon Y.L., Afford S.C., Mergental H., Perera M.T.P.R., Stephenson B.T.F., Fry L., Attard J., Barton D., Toogood G.J., Isaac J.R. (2019). Combined hypothermic and normothermic machine perfusion improves functional recovery of extended criteria donor livers. Liver Transpl..

[10] van Rijn R., Schurink I.J., de Vries Y., van den Berg A.P., Cortes Cerisuelo M., Darwish Murad S., Erdmann J.I., Gilbo N., de Haas R.J., Heaton N. (2021). Dual hypothermic oxygenated machine perfusion in liver transplants donated after circulatory death: A randomized clinical trial. Nat. Commun..

[11] Spada M., Angelico R., Riva S., Grimaldi C., Silvestri T., Giovannelli L., Gazia C., Francalanci P., Saffioti M.C., Rigamonti A. (2019). Hope for hypothermic machine perfusion in extended criteria liver donors. Transpl. Int..

[12] Schlegel A., Graf R., Clavien P.A., Dutkowski P., Mullhaupt B., Clavien P.A., de Rougemont O., Oberkofler C.E., Raptis D.A., Petrowsky H. (2018). The UK DCD Risk Score: A new proposal to define futility in donation-after-circulatory-death liver transplantation. J. Hepatol..

[13] Kogure K., Ishizaki M., Shimada M., Mizumoto M., Morioka D., Tanaka M., Sugiura T., Kawasaki S., Yokoi H., Nishizawa T. (2000). Anatomic variation of the biliary ducts, portal and hepatic veins as revealed by 3-dimensional venous and biliary tract imaging. Surg. Radiol. Anat..

[14] Sano K., Makuuchi M., Miki K., Maema A., Sugawara Y., Imamura H., Matsunami H., Takayama T., Kawasaki S., Hashimoto T. (2002). Criteria for reconstruction of the middle hepatic vein in living donor liver transplantation. Liver Transpl..

[15] Schlegel A., Muller X., Kalisvaart M., Muellhaupt B., Perera M.T.P.R., Isaac J.R., Clavien P.A., Muiesan P., Dutkowski P. (2019). Outcomes of DCD liver transplantation using organs treated by hypothermic oxygenated perfusion before implantation. J. Hepatol..

[16] Adam R., Karam V., Cailliez V., O’Grady J.G., Mirza D., Cherqui D., Klempnauer J., Salizzoni M., Pratschke J., Jamieson N. (2021). Evolution of indications and results of liver transplantation in Europe: A report from the European Liver Transplant Registry (ELTR). J. Hepatol..

[17] Nasralla D., Coussios C.C., Mergental H., Akhtar M.Z., Butler A.J., Ceresa C.D.L., Chiocchia V., Dutton S.J., García-Valdecasas J.C., Heaton N. (2018). A randomized trial of normothermic preservation in liver transplantation. Nature.

[18] Watson C.J.E., Hunt F., Messer S., Currie I., Large S., Sutherland A., Crick K., Wigmore S.J., Fear C., Cornateanu S. (2018). Observations on the ex situ perfusion of livers for transplantation. Am. J. Transplant..

[19] Schlegel A., Graf R., Clavien P.A., Dutkowski P., Kron P., de Rougemont O., Muiesan P., Perera M.T.P.R., Isaac J.R., Mergental H. (2019). Hypothermic oxygenated perfusion (HOPE) protects from biliary injury in a rodent model of DCD liver transplantation. J. Hepatol..

[20] van Rijn R., Karimian N., Matton A.P.M., Burlage L.C., Westerkamp A.C., van den Berg A.P., de Kleine R.H.J., de Boer M.T., Lisman T., Porte R.J. (2017). Dual hypothermic oxygenated machine perfusion in liver transplants donated after circulatory death. Br. J. Surg..

[21] Schlegel A., Muller X., Mueller M., Stepanova A., Kron P., de Rougemont O., Muiesan P., Clavien P.A., Galkin S., Camara R. (2020). Machine perfusion approaches in liver transplantation with special focus on hypothermic oxygenated perfusion. Expert. Rev. Gastroenterol. Hepatol..

[22] Brüggenwirth I.M.A., van Leeuwen O.B., de Vries Y., Dou L., Whitehouse G., Mueller M., Darwish Murad S., de Meijer V.E., Fujiyoshi M., Neil D.A.H. (2020). Biomarkers and bile duct injury in human liver transplantation: Translational research on the path to preventive medicine. Liver Transpl..

[23] Noujaim H.M., de Ville de Goyet J., Parente A., Bening C., Davenport M., Rela M., Heaton N.D., Tredger J.M., Dhawan A., Baker A. (2018). Biliary strictures following liver transplantation: Past, present and preventive strategies. Liver Transpl..

[24] de Vries Y., Matton A.P.M., Nijsten M.W.N., Werner M.J.M., van den Berg A.P., de Boer M.T., Pirenne J., Porte R.J., van Leeuwen O.B., Lisman T. (2019). Metabolic zonation of periportal and pericentral regions in dynamic cold storage/machine perfusion of porcine livers. Am. J. Transplant..

[25] Czigany Z., Pratschke J., Froněk J., Guba M., Schöning W., Raptis D.A., Andrassy J., Kramer M., Strnad P., Tolba R.H. (2019). Hypothermic oxygenated machine perfusion reduces early allograft injury and improves post-transplant outcomes in extended criteria donation liver transplantation from donation after brain death: Results from a multicenter randomized controlled trial. Ann. Surg..

[26] Jochmans I., Brat A., Davies L., Hendricks P., Monbaliu D., Pirenne J., Watson C.J.E., Porte R.J., van Rijn R., de Vries Y. (2020). End-ischemic dual hypothermic oxygenated machine perfusion preserves hepatobiliary function: A multicenter clinical trial. JHEP Rep..

[27] Yoon Y., Schwartz M.E., Tabrizian P., Heptulla R.A., Florman S.S., Roayaie S., Facciuto M., Gunson B.K., Mergental H., Perera M.T.P.R. (2021). Technical aspects of ex situ liver splitting for two adult recipients: A comparative analysis of anterior versus posterior approaches. Liver Transpl..

[28] Broering D.C., Wilms C., Bok P., Fischer L., Mueller L., Hillert C., Kim J.S., Sterneck M., Rogiers X., Lohse A.W. (2005). Splitting of the middle hepatic vein in full-right full-left splitting of the liver. Liver Transpl..

[29] Cillo U., Rossi R.E., Lauterio A., De Carlis L., Burra P., McCormack L., Colledan M., Salizzoni M., Romagnoli R., Citterio D. (2024). Full-left/Full-right Liver Splitting With Middle Hepatic Vein and Caval Partition During Dual Hypothermic Oxygenated Machine Perfusion. Transplantation.

[30] Mabrut J.Y., Schneck A.S., Lesurtel M., Mohkam K., Muller X., Dokmak S., Faitot F., Dondero F., Ciacio O., Sa Cunha A. (2021). Ex Vivo Liver Splitting and Hypothermic Oxygenated Machine Perfusion: Technical Refinements of a Promising Preservation Strategy in Split Liver Transplantation. Transplantation.

[31] Eshmuminov D., Becker D., Bautista Borrego L., Hefti M., Schuler M.J., Hagedorn C., Muller X., Mueller M., Onder C., Graf R. (2020). Meta-analysis of the clinical application of normothermic and hypothermic machine perfusion in liver transplantation. Liver Transpl..

[32] Spada M., Angelico R., Grimaldi C., Francalanci P., Saffioti M.C., Rigamonti A., Basile U., Maggiore G., Colledan M., Pinelli D. (2020). The New Horizon of Split-Liver Transplantation: Ex Situ Liver Splitting During Hypothermic Oxygenated Machine Perfusion. Liver Transpl..

[33] Thorne A.M., van Leeuwen O.B., de Vries Y., Nijsten M.W.N., de Boer M.T., de Kleine R.H.J., Leuvenink H.G.D., de Meijer V.E., Porte R.J., van Rijn R. (2021). Ex Situ Dual Hypothermic Oxygenated Machine Perfusion for Human Split Liver Transplantation. Transplant. Direct..

[34] Rossignol G., Muller X., Mohkam K., Lacaze L., Barbieux K., Julien C., Lesurtel M., Mabrut J.Y., Collardeau-Frachon S., McLin V.A. (2022). Full left/full right liver graft ex situ split during hypothermic oxygenated perfusion. Pediatr. Transplant..

[35] Buis C.I., Geuken E., Visser D.S., Kuipers F., Haagsma E.B., Verkade H.J., Hulstaert P.F., Gouw A.S.H., van den Berg A.P., Porte R.J. (2019). Nonanastomotic biliary strictures after liver transplantation, part 1: Radiological features and risk factors for early vs. late presentation. Liver Transpl..

[36] Rogiers X., Malago M., Gawad K., Jauch K.W., Olausson M., Knoefel W.T., Guckelberger O., Fischer L., Sterneck M., Broering D.C. (1996). In situ splitting of cadaveric livers. The ultimate expansion of a limited donor pool. Ann. Surg..

[37] Krendl F.J., Cardini B., Oberhuber R., Fodor M., Sucher R., Hautz T., Öfner D., Schneeberger S., Margreiter C., Troppmair J. (2024). Normothermic Liver Machine Perfusion and Successful Transplantation of Split Liver Grafts: From Proof of Concept to Clinical Implementation. Transplantation.

[38] Mergental H., Laing R.W., Kirkham A.J., Perera M.T.P.R., Boteon Y.L., Attard J., Barton D., Curbishley S., Wilkhu M., Neil D.A.H. (2022). Evaluation of viability of marginal donor livers by transplanting organs after evaluation with normothermic machine perfusion: Results of a multicenter, prospective trial. J. Hepatol..

[39] Karangwa S., Panayotova G., Dutkowski P., Porte R.J., Guarrera J.V., Schlegel A. (2020). Hypothermic machine perfusion in liver transplantation. Int. J. Surg..

[40] Panaro F., Benedetti E., Pineton de Chambrun G., Habibeh H., Leon P., Bouyabrine H., Herrero A., Navarro F. (2018). Indocyanine green fluorescence angiography during liver and pancreas transplantation: A tool to integrate perfusion statement’s evaluation. Hepatobiliary Surg. Nutr..

[41] Clavien P.A., Lesurtel M., Bossuyt P.M., Gok B., O’Mahony L., Reeves H.L., Robles R., Figueras J., Jaurrieta E., Navasa M. (2022). Current status and future perspectives in liver transplantation: From splitting to artificial organs. Nat. Rev. Gastroenterol. Hepatol..

